# Polymorphic genes of detoxification and mitochondrial enzymes and risk for progressive supranuclear palsy: a case control study

**DOI:** 10.1186/1471-2350-13-16

**Published:** 2012-03-17

**Authors:** Lisa F Potts, Alex C Cambon, Owen A Ross, Rosa Rademakers, Dennis W Dickson, Ryan J Uitti, Zbigniew K Wszolek, Shesh N Rai, Matthew J Farrer, David W Hein, Irene Litvan

**Affiliations:** 1 Department of Anatomical Sciences and Neurobiology, University of Louisville, Louisville, KY, USA; 2 Department of Bioinformatics and Biostatistics, University of Louisville, Louisville, KY, USA; 3 Department of Neuroscience, Mayo Clinic Jacksonville, Jacksonville, FL, USA; 4 Department of Neurology, Mayo Clinic Jacksonville, Jacksonville, FL, USA; 5 J.G. Brown Cancer Center, University of Louisville, Louisville, KY, USA; 6 Department of Medical Genetics, Centre of Applied Neurogenetics, Brain Research Centre, University of British Columbia, Vancouver, British Columbia, Canada; 7 Department of Pharmacology and Toxicology, University of Louisville, Louisville, KY, USA; 8 Department of Neurology, University of Louisville, Louisville, KY, USA; 9 Department of Neurosciences, University of California San Diego, La Jolla, CA, USA

**Keywords:** Progressive supranuclear palsy (PSP), *N*-acetyltransferase 2 (NAT2), Tauopathy, Single nucleotide polymorphisms (SNPs), Parkinson's disease (PD)

## Abstract

**Background:**

There are no known causes for progressive supranuclear palsy (PSP). The *microtubule associated protein tau *(*MAPT) *H1 haplotype is the major genetic factor associated with risk of PSP, with both oxidative stress and mitochondrial dysfunction also implicated. We investigated whether specific single nucleotide polymorphisms (SNPs) in genes encoding enzymes of xenobiotic detoxification, mitochondrial functioning, or oxidative stress response, including *debrisoquine 4-hydroxylase, paraoxonase 1 *and *2, N-acetyltransferase 1 *and *2 (NAT2), superoxide dismutase 1 *and *2, and PTEN-induced putative kinase *are associated with PSP.

**Methods:**

DNA from 553 autopsy-confirmed Caucasian PSP cases (266 females, 279 males; age at onset 68 ± 8 years; age at death 75 ± 8) from the Society for PSP Brain Bank and 425 clinical control samples (197 females, 226 males; age at draw 72 ± 11 years) from healthy volunteers were genotyped using Taqman PCR and the SequenomiPLEX Gold assay.

**Results:**

The proportion of NAT2 rapid acetylators compared to intermediate and slow acetylators was larger in cases than in controls (OR = 1.82, p < 0.05). There were no allelic or genotypic associations with PSP for any other SNPs tested with the exception of *MAPT *(p < 0.001).

**Conclusions:**

Our results show that NAT2 rapid acetylator phenotype is associated with PSP, suggesting that NAT2 may be responsible for activation of a xenobiotic whose metabolite is neurotoxic. Although our results need to be further confirmed in an independent sample, NAT2 acetylation status should be considered in future genetic and epidemiological studies of PSP.

## Background

Progressive supranuclear palsy (PSP) is the most common atypical parkinsonian disorder. Classically, patients present with progressive postural instability and falls followed by slow and hypometric vertical saccades and eventually vertical supranuclear gaze palsy.

Neuropathologically, PSP is characterized by deposits of four-repeat microtubule associated protein tau (encoded by the *MAPT *gene) aggregates in neurons and glia of the basal ganglia and brain-stem [1]. Additionally, there is mitochondrial dysfunction, decreased ATP levels and inflammation in the brains of PSP patients [2-4]. The *MAPT *H1 haplotype has been consistently reported to be associated with PSP; however, it is also common in the general population, suggesting that gene-gene or gene-environment interactions are likely required for the development of this disease [5,6]. Recently, *MAPT H1 *was also associated with risk of Parkinson's disease (PD) suggesting shared pathways of disease [7]. Early-onset PD and PSP can present with a similar phenotype and be misdiagnosed, supporting common links between the two disorders. The product of *PTEN-induced putative kinase (PINK1, PARK6)*, associated with early-onset PD, is involved in mitochondrial respiration and protection from oxidative damage, which are pathways that have also been linked to risk of PSP [8-13]. PINK-1 polymorphisms are also associated with PD and it acts in conjunction with parkin to regulate mitochondrial functioning. Although the mechanisms by which PINK1 acts are not fully understood; research suggests that it is crucial for healthy mitochondrial respiration and ATP production [8]. Considering the role of PINK1 in mitochondrial functioning along with its previous links to PD, specific PINK1 SNPs were included in this study to determine if there is also an association with PSP.

Consumption of annonaceous fruit and teas, which contain mitochondrial inhibitors, has been associated with an atypical parkinsonian disorder similar to PSP in the French West Indies [14,15]. Considering that mitochondrial impairment is observed in PSP brains, mitochondrial complex-1 inhibitors and other chemical neurotoxins, such as organophosphates, are hypothesized as risk factors for PSP [16-18]. These and other potentially toxic compounds are metabolized by the products of several genes: *debrisoquine 4-hydroxylase (CYP2D6), paraoxonase (PON) 1 *and *2, N-acetyltransferase (NAT) 1 *and *2*, and *superoxide dismutase (SOD) 1 *and *2 *[10-13,19-22]. CYP2D6 is found in the brain and is involved in metabolism of MPTP, herbicides (paraquat) and organophosphate pesticides [11,12]. Reduced in 5-10% of Caucasians, genetic polymorphisms of this enzyme have been widely studied in PD and results suggest that there is an association of the poor metabolizer phenotype with disease development [23,24]. Moreover, the combination of pesticide exposure and CYP2D6 poor metabolizer phenotype doubles PD risk [11,20]. PON1 hydrolyzes phosphoric acid esters, organophosphates and aromatic carboxylic acid esters and blocks the formation of free radicals. With low PON1 activity, these pesticides are not metabolized and the cell is subject to increased oxidative stress [19]. The PON 1 M allele, which is correlated with decreased protein levels, has been shown to be associated with PD [25,26] and the M/M genotype was recently reported to be associated with early onset PD [27]. Additionally, decreased PON1 activity was overrepresented in PD patients from agriculturally exposed areas [19]. NAT1 and NAT2 are involved in the biotransformation of drugs and environmental toxins (xenobiotics) [28]. These enzymes transfer the acetyl group from acetyl-coenzyme A (acetyl CoA) to an amino group on aromatic amines and hydrazine compounds. In addition, following N-hydroxylation, they can further activate xenobiotics via O-acetylation [29]. There are a number of SNPs reported in NAT1 and NAT2, which lead to slow and rapid acetylator phenotypes. The acetylation status of an individual might determine how they respond to xenobiotic exposures, therefore presenting the NAT genes as candidates for gene-environment interaction studies. The slow acetylator phenotype is reported to be associated with PD, but inconsistent results warrant further investigation [30-34]. SOD is an important antioxidant enzyme, which converts superoxide anions (O_2_-) to hydrogen peroxide (H_2_O_2_). Considering the antioxidant properties of the enzyme, polymorphisms resulting in decreased SOD activity would be expected to have detrimental effects on the cell; however, recent studies suggest the opposite is true [35-37]. The mechanism behind this gain of function toxicity remains unknown, but it is proposed to be a result of either 1) disrupting the balance of O_2_- and H_2_O_2_, or 2) self-aggregation. Numerous SOD polymorphisms have been found to be associated with amyotrophic lateral sclerosis (ALS) [38], and may play a role in PD and AD pathogenesis [39].

To determine if genetic polymorphisms in toxicant metabolism increases risk for developing PSP, we investigated associations between PSP and specific single nucleotide polymorphisms (SNPs) in the aforementioned genes.

## Methods

### Sample

DNA samples from 545 autopsy-confirmed PSP cases collected between 1993 and 2008 at the PSP Society Brain Bank were included [40]. All cases were from the US and Canada. Control DNA samples (n = 426) were randomly selected from an existing repository of control samples at the Mayo clinic, Jacksonville. All controls were healthy spouses or caregivers of patients at the Mayo Clinic in Jacksonville, FL and free from neurological disorders. All samples were from adults over the age of 33 (see Table 1 for demographic information). Institutional review board (IRB)-approved protocols, including informed consent, were followed to obtain all DNA samples.

**Table 1:** Characteristics of PSP Cases and Controls

	**Case (N = 545)**^**a**^		Control (N = 426)		
	n	%	n	%	p
**Gender**					0.490
Female	266	49	197	47	
Male	279	51	226	53	
**Race**					--
Caucasian	545	100	426	100	
**Age**^**b**^					**< 0.001**
Median(Min, Max)	76(44,98)		73(33,92)		
Mean(SD)	75.4(8.0)		72(10.7)		
**Clinical Diagnosis**					
PSP	468	87			
Other^c^	68	13			
**Falls**					
Yes	482	99			
No	3	1			
**Dementia**					
Yes	278	82			
No	62	18			
**Eye Mov. Abn**.					
Yes	349	96			
No	13	4			
**Rigidity**					
Yes	374	98			
No	9	2			
**Instability**					
Yes	454	99			
No	4	1			
**Dysarthria**					
Yes	370	97			
No	10	3			
**Dysphagia**					
Yes	352	92			
No	31	8			
**AD Path**.					
Yes	50	9			
No	495	91			
**Family History**					
Yes	322	73			
No	121	27			

^a^Ns of cases for characteristics including and following "clinical diagnosis" may not total 545 as information for these variables was not available for every case.% represent% of those with data. ^b^Age represents age at death for cases and age at blood draw for controls. ^c^Other clinical diagnoses included Alzheimer disease, Parkinson's disease, corticobasal degeneration, multiple system atrophy, Parkinson's disease with dementia, vascular dementia, progressive non-fluent aphasia and dementia. Abbreviations: p = p-value (chi-squared), Eye Mov. Abn. = eye movement abnormalities, AD Path. = presence of coexisting Alzheimer's pathology

### Genotyping

Within 48 hours of collection, DNA was extracted by standard protocols and stored at -80°C until used. *NAT1 *(rs4987076, rs5030839, rs4986782, rs1057123, and rs15561) and *NAT2 *(rs1208, rs1801279, rs1801280, rs1799929, rs1799930, rs1799931, and rs1041983) genotyping was performed using Taqman PCR methodology on an ABI Prism 7700 sequence detection system as previously published [41,42]. All other genotyping was performed on a Sequenom Mass Array iPLEX platform using the Gold Assay (San Diego, CA) as described previously [43] (see Table 2 for rs numbers). Primer sequences are available upon request. The rs numbers tested here also included in the recent GWAS on PSP are rs1043424, rs662, rs7493, rs1801280, rs1799930, rs1799931, rs1799929, and rs1041983 [44].

**Table 2:** Case-control comparison of SNP genotypes

	Total n	Total n	n-controls			n-cases						p		q
SNP rs marker (gene name)	**Controls (freq)**^**a**^	**Cases (freq)**^**a**^	0	1	2	0	1	2	OR	CI	trend	**recess**.	**dom**.	Holm
rs1135840	395	500	133	183	79	145	240	115	1.14	0.94-1.37	0.174	0.367	0.188	1
(*CYP2D6*)	0.44	0.56	0.34	0.56	0.2	0.39	0.5	0.23						
rs3738136	412	521	375	36	1	469	48	4	1.11	0.73-1.7	0.625	0.338	0.78	1
(*PINK*-*1*)	0.44	0.56	0.91	0.19	0	0.9	0.19	0.01						
rs1043424	414	520	217	165	32	281	208	31	0.89	0.72-1.1	0.27	0.19	0.478	1
(*PINK*-*1*)	0.44	0.56	0.52	0.4	0.18	0.54	0.4	0.16						
rs2234694	412	521	372	38	2	478	41	2	0.84	0.54-1.28	0.411	0.813	0.408	1
(*SOD1*)	0.44	0.56	0.9	0.19	0	0.92	0.18	0						
rs4880	413	523	107	206	100	116	264	143	1.14	0.95-1.38	0.157	0.22	0.279	1
(*SOD2*)	0.44	0.56	0.36	0.5	0.24	0.22	0.5	0.37						
rs662	414	521	211	160	43	256	217	48	1.03	0.85-1.26	0.757	0.65	0.497	1
(*PON1*)	0.44	0.56	0.51	0.49	0.1	0.59	0.42	0.09						
rs854560	412	521	181	176	55	213	242	66	1.02	0.84-1.23	0.875	0.661	0.606	1
(*PON1*)	0.44	0.56	0.44	0.43	0.13	0.41	0.56	0.13						
rs705381	409	523	231	148	30	293	201	29	0.98	0.79-1.21	0.845	0.355	0.831	1
(*PON1*)	0.44	0.56	0.6	0.46	0.11	0.66	0.48	0.16						
rs7493	413	523	237	148	28	315	181	27	0.9	0.73-1.12	0.349	0.265	0.535	1
(*PON2*)	0.44	0.56	0.67	0.46	0.17	0.6	0.45	0.15						
rs12026	412	521	237	147	28	314	180	27	0.9	0.73-1.12	0.364	0.267	0.558	1
(*PON2*)	0.44	0.56	0.68	0.46	0.17	0.6	0.45	0.15						
**rs1052553**	**406**	**324**	**231**	**148**	**27**	**281**	**40**	**3**	**0.23**	**0.16-0.32**	**< 0.001**	**0.001**	**< 0.001**	**< 0.001**
(*MAPT*)	0.56	0.44	0.67	0.46	0.17	0.97	0.12	0.01						

Logistic regression trend test comparing genotype differences between cases and controls, adjusted for age (age at death for cases, age at draw for controls). ^a^Frequency = number of samples in a particular variable category (i.e. "cases" or "0, 1, 2") divided by the total n for that variable (i.e. "cases + controls" or "0+1+2"). "0" represents common allele homozygous genotype for SNP tested, 1 = heterozygote, 2 = minor allele homozygous. OR = odds ratios for increase in the number of alleles (i.e. from 0 to 1 or 1 to 2); CI = 95% confidence intervals for ORs; p = p-values reported for trend test as well as for recessive (recess.) and dominant (dom.) models;q = q-values for multiple testing adjustment using Holm's method.

### Data analysis

Statistical analyses were performed using R software (R Development Core Team 2009). Chi-squared, Fisher's exact, student *t*-test, or Wilcoxon rank sum analyses were used to test for differences in demographic variables between cases and controls. For each iPLEX SNP variable, the Cochran-Armitage and chi-squared tests were used to test additive, dominant, and recessive genetic models. In addition, logistic regression was used to test these same genetic models while adjusting for significant demographic variables (i.e. age). Logistic regression models were also used to determine whether specific *NAT1 *or *NAT2 *genotypes or NAT2 phenotypes were associated with PSP. NAT2 phenotypes may be accurately assigned according to genotype [22]; therefore, NAT2 analysis was initially restricted to phenotypic evaluation, which was followed by genotypic analysis. Overall significance of the associations was determined using the omnibus chi-squared test for the model. If the omnibus chi-squared test was not significant, then individual genotypes were not considered significant even if the associated p-value (p) was < 0.05. Odds ratios (OR), 95% confidence intervals (CI) and p-values were determined for each variable. Associations with p < 0.05 were considered significant. Based on the outcome of the primary analysis, *t*-test or Wilcoxon rank sum test was applied to determine whether means/medians were different between NAT2 phenotypes for age at onset, age at death or disease duration in cases. *NAT2 *genotype, *NAT1 *genotype and iPLEX SNP associations were all tested independently each with either a large number of groups or a low number of tests; nevertheless, when p-values were less than 0.05, adjustments were made for multiple testing using the Holm correction [45]. NAT2 phenotype tests were modeled independently from SNP analyses. Furthermore, while multiple SNPs were determined to input the phenotypes, only two phenotypes were compared (i.e. rapid versus slow/intermediate), therefore no multiple testing correction was needed as previously described for testing the NAT2 phenotype association with colorectal cancer [46].

## Results

On average, cases were older than controls at sample collection time (Table 1, p < 0.001), with age at collection time for PSP cases being age at death. Trend analysis of the iPLEX SNPs showed no between-group differences in genotypes (Table 2), with the exception of rs1052553 (*MAPT H1 *OR = 4.35, CI = 3.08-6.25, p < 0.001), which is a known association [47]. Each marker was confirmed to be in Hardy-Weinberg equilibrium in controls. Minor allele frequencies (MAFs) for rs numbers 1043424 and 705381 were higher in both our PSP and control populations compared to that reported for the general (Caucasian) population. For rs numbers 4880 and 1052553 only the PSP sample differed from the general population (Table 3). There were no between-group differences for *NAT1 *genotypes (Table 4). NAT2 slow and intermediate phenotypes did not differ between groups (p = 0.96), thus these groups were combined and compared against the rapid phenotype for further analyses. Phenotypic analysis showed cases had a significantly higher proportion of NAT2 rapid acetylators (OR = 1.82, CI = 1.05-3.28, p = 0.037) compared to intermediate and slow (Table 5). The omnibus chi-squared test for NAT2 genotypes was not significant (Table 6). Since NAT2 rapid phenotype was associated with PSP, rank sum analyses were used to determine whether NAT2 acetylation status predicted either age at onset or disease duration. NAT2 phenotype was not associated with age at onset or age at death. For disease duration the overall test was also not significant; however, individual pairwise comparisons for disease duration using a *t*-test (unequal variances, Table 7) corroborated results for association of NAT2 rapid phenotype with disease (Table 5). For example, mean disease duration was shorter for rapid NAT2 phenotype (6.6 yrs.) compared to slow (7.5 yrs. p = 0.025).

**Table 3:** Allele Frequencies of SNPs vs. General Population

	Allele	MAF	Controls			q	Cases			
Marker	Mn/Mj	**pop**^**a**^	MAF	CI	p	Holm	MAF	CI	p	q
rs1135840	G/C	0.43	0.432	0.40-0.47	0.954	1	0.47	0.44-0.50	0.012	0.081
rs3738136	A/G	0.06	0.046	0.03-0.06	0.109	0.543	0.054	0.04-0.07	0.432	1.000
rs1043424	C/G	0.34	0.277	0.25-0.31	< 0.001	0.001	0.26	0.23-0.29	< 0.001	< 0.001
rs2234694	C/A	0.042	0.051	0.04-0.07	0.231	0.925	0.043	0.03-0.06	0.91	1.000
rs4880	C/T	0.45	0.492	0.46-0.53	0.018	0.145	0.526	0.50-0.56	< 0.001	< 0.001
rs662	G/A	0.33	0.297	0.27-0.33	0.048	0.337	0.3	0.27-0.33	0.046	0.273
rs854560	T/A	0.38	0.347	0.31-0.38	0.056	0.337	0.359	0.33-0.39	0.171	0.854
rs705381	T/C	0.18	0.254	0.23-0.29	< 0.001	< 0.001	0.248	0.22-0.28	< 0.001	< 0.001
rs7493	G/C	0.24	0.247	0.22-0.28	0.668	1	0.225	0.20-0.25	0.261	1.000
rs12026	G/C	0.24	0.246	0.22-0.28	0.699	1	0.225	0.20-0.25	0.258	1.000
rs1052553	G/A^b^	0.21	0.249	0.22-0.28	0.008	0.068	0.071	0.05-0.09	< 0.001	< 0.001

MAFs of studied population (control/case) compared with the general population (MAF pop) using test of proportions. ^a^Minor allele frequencies listed on NCBI from CEU data (CEU = Utah residents with north and western European ancestry). ^b^Note G = H2, A = H1. Mn/Mj -Minor Allele/Major Allele; MAF = Minor Allele Frequency; CI = 95% confidence intervals for determined MAF for noted population (i.e. control or case); p = p-value (Chi-squared); q = q-values for multiple testing adjustment using Holm's method.

**Table 4:** Case-control comparison of NAT1 genotypes

	Controls(N = 426)		Cases (N = 545)				
NAT1 Genotype	n	%	n	%	OR	CI	p
NAT1*4/*4	211	50.8	268	49.7	1		
NAT1*10/*11A	4	1.0	5	0.9	1.03	0.26-4.35	0.967
NAT1*10/*10	14	3.4	22	4.1	1.22	0.61-2.52	0.583
NAT1*4/*3	13	3.1	23	4.3	1.50	0.74-3.14	0.271
NAT1*4/*14A or *10/*14B	13	3.1	19	3.5	1.25	0.60-2.66	0.552
NAT1*10/*14A	4	1.0	6	1.1	1.12	0.31-4.45	0.863
NAT1*4/*10	138	33.3	165	30.6	0.94	0.70-1.26	0.677
NAT1*4/*11A or *3/*11B	13	3.1	21	3.9	1.21	0.59-2.55	0.607
NAT1*10/*15	0	0.0	1	0.2	--	--	--
NAT1*11A/*14A	0	0.0	1	0.2	--	--	--
NAT1*3/*10	2	0.5	4	0.7	1.65	0.32-12.10	0.567
NAT1*3/*14A	0	0.0	1	0.2	--	--	--
NAT1*4/*11B	0	0.0	1	0.2	--	--	--
NAT1*4/*15	3	0.7	2	0.4	0.37	0.05-2.39	0.298
Missing	11		6				

Logistic regression analysis of individual NAT1 genotypes, adjusted for age. NAT1*4/*4 used as reference. Overall chi-squared p = 0.99 (likelihood ratio test). Genotypes with 10 or less counts in either group (case or control) were not included in overall test of significance. OR = odds ratio, CI = 95% confidence intervals for ORs, p = p-value

**Table 5:** Comparisons Between NAT2 Phenotypes

	Controls(N = 426)		Cases(N = 545)					
NAT2 Phenotype	n	%	n	%	OR	CI	p	q
**Slow**	241	56.6	299	54.9	1	-	-	-
**Intermediate**	161	37.8	198	36.4	0.993	0.76-1.31	0.959	0.959
**Rapid**	19	4.5	42	7.7	1.82	1.04-3.30	0.042a	0.084

Logistic regression analysis of individual NAT2 phenotypes, adjusted for age OR = odds ratio, CI = 95% confidence intervals for ORs, p = p-value, q = q-values for multiple testing adjustment using Holm's method. ^a^When intermediate and slow phenotypes are combined (due to p = 0.96) and used as reference, the p-value for rapid is 0.037, and no multiple testing correction is needed since it is a single hypothesis

**Table 6:** Case-control comparison of NAT2 genotypes

	Controls(N = 426)		Cases (N = 545)					
NAT2 Genotype (phenotype)	n	%	n	%	OR	CI	p	q
NAT2*4/*4 (rapid)	19	4.5	42	7.7	1			
NAT2*4/*5(intermediate)	**108**	**25.4**	**116**	**21.3**	**0.49**	**0.26-0.88**	**0.021**	0.168
NAT2*4/*6 (intermediate)	49	11.5	69	12.7	0.6	0.30-1.16	0.133	0.532
NAT2*4/*7 (intermediate)	4	0.9	13	2.4	1.43	0.43-5.65	0.576	1
NAT2*5/*6 (slow)	106	24.9	130	23.9	0.55	0.29-0.99	0.053	0.318
NAT2*5/*7 (slow)	5	1.2	10	1.8	0.86	0.26-3.10	0.809	1
NAT2*6/*6(slow)	**42**	**9.9**	**47**	**8.6**	**0.46**	**0.23-0.92**	**0.029**	0.203
NAT2*6/*7 (slow)	5	1.2	7	1.3	0.56	0.15-2.21	0.394	1
NAT2*5/*5 (slow)	82	19.2	104	19.1	0.58	0.31-1.07	0.086	0.430
NAT2*5/*14 (slow)	1	0.2	0	0	--	--	--	
NAT2*6/*14 (slow)	0	0	1	0.2	--	--	--	
Missing	5		6		--	--	--	

Logistic regression analysis of individual NAT2 genotypes, adjusted for age. NAT2*4/*4 used as reference. OR = odds ratio; CI = 95% confidence intervals for ORs; p = p-value; q = q values for multiple testing adjustment using Holm's method. Overall chi-squared p = 0.25 (likelihood ratio test). Genotypes with 10 or less counts in either group (case or control) were not included in overall test of significance

**Table 7:** Survival of PSP cases by NAT2 phenotype

NAT2 Phenotype	Median(Min, Max)	Mean(SD)	p-values
Age at Onset			
Rapid	68.5(51,85)	68.5(8.5)	> 0.05
Intermediate	68(41,89)	68.1(8.6)	> 0.05
Slow	68(47,90)	67.9(8.1)	--
Age at Death			
Rapid	75.5(58,89)	75.3(7.9)	> 0.05
Intermediate	76(44,98)	75.3(8.3)	> 0.05
Slow	76(53,95)	75.4(7.9)	--
Disease Duration (yrs.)			
Rapid^a^	6(2,12)	6.6(2.2)	0.025, 0.078, 0.028
Intermediate^b^	7(2,31)	7.4(3.6)	0.675
Slow	7(0,27)	7.5(3.3)	--

Pairwise comparisons of age at onset, age at death, and disease duration by NAT2 phenotype (*t*-test with unequal variances). ^a^p-values = rapid vs. slow, rapid vs. intermediate, rapid vs. slow + intermediate, respectively. ^b^p-value = slow vs. intermediate. Overall p-values from Wilcoxon rank sum tests were all > 0.1

## Discussion

Our primary analysis revealed that none of the iPLEX SNPs was proportionally different between cases and controls except for *MAPT *rs1052553, which is a known association. On the other hand, significant differences were detected when comparing MAFs of cases with reported MAFs for the general population. There were no differences in *NAT1 *or *NAT2 *genotypes between cases and controls. NAT2 rapid acetylator phenotype was more frequent in PSP cases than controls while intermediate and slow acetylator phenotypes were less frequent in cases.

Although trend analysis did not show differences between cases and controls for the iPLEX SNPs (i.e. except for rs1052553), cases did differ from the general population (CEU) in some MAFs. Of particular interest is *SOD2 *rs4880, which differed from the general population in cases, but not controls. Though not conclusive, this suggests a possible association of rs4880 with PSP. The *MAPT H1 *allele is known to be associated with PSP; however, it is the major allele. Consistent with previous studies, we found that *MAPT *genotype and MAFs differed between PSP cases, with the *H1 *allele conferring risk [6,44]. Furthermore, MAF comparisons indicate the *H2 *allele is protective, as it had a lower frequency in our cases compared to the general population (Table 3). Our results also suggest that NAT2 rapid acetylator status might increase risk for developing PSP. This is consistent with NAT2-catalyzed toxicant activation (perhaps via O-acetylation). Therefore, a higher rate of acetylation would result in a higher concentration of toxic metabolite in the system. NAT2 catalyzes the O-acetylation of N-arylhydroxylamines resulting in bioactivation [48].

This is an observational study, therefore more emphasis should be placed on the estimated odds ratio and precision of the confidence intervals rather than on p-values [49]. Nevertheless, these trends must be confirmed by additional studies. Our results did not provide statistical evidence for an effect of NAT2 phenotype on onset age, age at death or disease duration. In accord with our finding that NAT2 rapid phenotype is more frequent in cases than controls, pairwise comparisons did show a trend supporting a potential link between rapid phenotype and shorter disease duration (Table 7). It is important to note that this particular analysis may have been underpowered for detecting differences in the outcome parameters since the lack of disease onset and duration information for many cases substantially decreased the sample size.

Our findings are noteworthy as NAT2*4, which confers the rapid phenotype, was designated originally as the "wild-type" allele http://louisville.edu/medschool/pharmacology/nat/ since it is common among many ethnic groups other than Europeans or Caucasians [29]. Although the frequency of NAT2*4 is not as common among Caucasians (which is the group analyzed in our study), this association may still be similar to the *MAPT H1 *haplotype association with PSP (i.e. *MAPT H1 *is associated with increased PSP risk, but is also very common in the general population with a frequency of 0.78) [6]. Therefore, even though our results suggest the rapid acetylator phenotype increases risk for PSP, this is only one of potentially numerous factors that converge to determine individual risk for disease. On the other hand, our finding is contrary to recent findings that NAT2 rapid acetylator genes enhance the protective effect of smoking in PD (De Palma et al. 2010) and reports suggesting that the NAT2 slow acetylator phenotype increases risk for PD [50-52]. PSP is a tauopathy and PD is a synucleinopathy, thus, these are two distinct diseases that may have distinct pathogenic mechanisms and risk factors [53]. There are varying reports of *NAT2 *polymorphisms associating with PD, PSP, and AD. While many suggest that slow alleles or phenotypes increase disease risk [31,32,50-52], others indicate increased risk with rapid or intermediate conferring genotypes and protection by slow alleles or genotypes [13,54]. Still others suggest there are no links between these diseases and *NAT *polymorphisms [18,30,33,55,56]. In view of these conflicting reports on the role of *NAT *genetic polymorphisms in neurodegeneration together with our results, additional studies are needed to determine whether *NAT *alleles or genotypes conferring rapid acetylation increase risk for neurodegenerative diseases or if the slow alleles/genotypes are protective or vice versa.

## Conclusions

The control series we used was more geographically confined than our PSP population and the CEU population from which the general population MAFs were derived. Interestingly, for some of the MAFs our control population differed from the general population. This could explain why our genotype comparisons between cases and controls were not significant. Therefore, MAF comparisons between our PSP sample and the general/CEU population augment our case-control analyses. The main strength of this study was the large sample of pathologically well-characterized PSP cases from a single center. On the other hand, the clinical information was not collected in a systematic or standardized manner and controls were clinical, not pathological controls. Considering that PSP is a relatively rare disease, a still larger sample size may be necessary to detect smaller, yet biologically significant differences and investigate interaction effects. Likewise, as 514 of the PSP cases analyzed here were also included in the GWAS, this finding should be confirmed in an independent cohort. Although these findings need to be replicated, this data provides useful information to guide future genetic studies on PSP as it indicates that NAT2 rapid acetylator status should be considered as a potential risk factor for PSP in studies investigating gene-gene and gene-environment interactions. Furthermore, our results are consistent with the recent genome-wide association study (GWAS) on PSP that did not find any associations with SNPs rs1043424, rs662, rs7493 or any individual *NAT2 *SNPs [44]. The *NAT2*rs numbers tested here and included in the recent GWAS on PSP are rs1801280, rs1799930, rs1799931, rs1799929, and rs1041983 [44]. Though we did not find an association with any individual *NAT2 *SNPs, when we used the SNPs to input NAT2 phenotype we observed a significant association between imputed rapid NAT2 acetylator phenotype and PSP. This result is important since this method of testing NAT2 phenotype association with disease has been shown to be more useful than looking at individual SNPs [57,58]. Thus, our study is quite different from the GWAS, and with respect to NAT2, much more powerful in terms of biological plausibility. Additionally, this study reveals the odds ratios and confidence intervals for a number of biologically relevant SNPs that have not been previously investigated in association studies on PSP. Our results provide support for the multiple-hit hypothesis and demonstrate the multifaceted nature of identifying risk factors for neurodegenerative diseases such as PSP.

## Competing interests

The authors declare that they have no competing interests. This research was conducted in accordance with institutional review board approved procedures.

## Authors' contributions

LFP participated in study conception and design, carried out NAT genotyping, assisted in data analysis and was primarily responsible for drafting the manuscript. ACC performed statistical analysis and assisted in data interpretation and manuscript preparation. OAR participated in study design and iPLEX genotyping and manuscript critique. RR helped with study design and DNA preparation. DWD, RJU and ZWK provided samples and were involved in manuscript review and critique. SNR assisted in statistical analysis and data interpretation. MJF participated in study design and manuscript critique. DWH participated in study design, data analysis, and manuscript critique. IL was responsible for study conception, design and manuscript review and critique.

## Pre-publication history

The pre-publication history for this paper can be accessed here:

http://www.biomedcentral.com/1471-2350/13/16/prepub
